# Using motion‐detection cameras to monitor foraging behaviour of individual butterflies

**DOI:** 10.1002/ece3.70032

**Published:** 2024-07-21

**Authors:** Denise Dalbosco Dell’Aglio, Owen W. McMillan, Stephen Montgomery

**Affiliations:** ^1^ Smithsonian Tropical Research Institute Panama City Panama; ^2^ School of Biological Science University of Bristol Bristol UK

**Keywords:** camera traps, feeding, foraging, Heliconiini, *Heliconius*, spatial pattern, temporal pattern

## Abstract

The activity of many animals follows recurrent patterns and foraging is one of the most important processes in their daily activity. Determining movement in the search for resources and understanding temporal and spatial patterns in foraging has therefore long been central in behavioural ecology. However, identifying and monitoring animal movements is often challenging. In this study we assess the use of camera traps to track a very specific and small‐scale interactions focused on the foraging behaviour of Heliconiini butterflies. Data on floral visitation was recorded using marked individuals of three pollen‐feeding species of *Heliconius* (*H. erato*, *H. melpomene* and *H. sara*), and two closely related, non‐pollen feeding species (*Dryas iulia* and *Dryadula phaetusa*) in a large outdoor insectary. We demonstrate that camera traps efficiently capture individual flower visitation over multiple times and locations and use our experiments to describe some features of their spatial and temporal foraging patterns. Heliconiini butterflies showed higher activity in the morning with strong temporal niche overlap. Differences in foraging activity between males and females was observed with females foraging earlier than males, mirroring published field studies. Some flowers were more explored than others, which may be explained by butterflies foraging simultaneously affecting each other's flower choices. Feeding was grouped in short periods of intense visits to the same flower, which we refer to as feeding bouts. *Heliconius* also consistently visits the same flower, while non‐*Heliconius* visited a greater number of flowers per day and their feeding bouts were shorter compared with *Heliconius*. This is consistent with *Heliconius* having more stable long‐term spatial memory and foraging preferences than outgroup genera. More broadly, our study demonstrates that camera traps can provide a powerful tool to gather information about foraging behaviour in small insects such as butterflies. © 2024 The Authors. Ecology and Evolution published by John Wiley & Sons Ltd.

## Graphical abstract

The use of camera traps is a powerful tool to gather information about foraging behaviour in butterflies. Our data reveal general trends such as temporal pattern of species activity that overlap, but with consistent differences in the daily activities of males and females. We observed short periods of intense visits to the same flower, with *Heliconius* consistently visiting the same flower, while non‐*Heliconius* visiting a greater number of flowers per day. Such data can provide important information about natural activity patterns, and potentially provide a context for experimental and diversity studies.

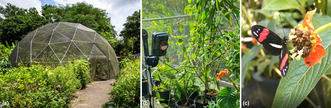

## 1 INTRODUCTION

When foraging, individual animals need to locate and consume resources, balancing the energy spent in this task with the energy gained from acquired resources. This balance depends on factors ranging from resource availability, competition, to predation risk (Stephens & Krebs, 2019). The measurement of animal foraging patterns is important to understanding fundamental behavioural strategies that reflect these trade‐offs, and can have applied use in conservation biology to understand the individual and population response to habitat fragmentation and biodiversity loss. However, identifying and monitoring animal movement during foraging is often challenging. Monitoring movements can involve manual annotations of observed behaviours, or mark and recapture data, which both require substantial human effort, or the use of technologies such as radar and GPS tracking which are costly and unavailable for many small animals. Some behavioural interactions are also difficult to observe and monitor through traditional methods due to their infrequency and unpredictability over time, and/or the requirement for invasive human observation over multiple sites.

Nonetheless, new studies monitoring animal movement are increasing in the field of ecological and conservation research, often through the use of modern and affordable camera traps (van Klink et al., 2022; Wägele et al., 2022). These cameras are positioned in strategic locations in a study site to capture recordings of individuals through motion‐triggering. Motion‐detection cameras have been used to gather large amounts of animal movement data and address a range of research questions including animal diversity and behaviour, from threatened big mammals, to nocturnal and shy species (Trolliet et al., 2014). Although these cameras usually monitor warm‐blooded animals, they can also provide a powerful tool to gather data on insects (Howard et al., 2021; Lihoreau et al., 2012; Naqvi et al., 2022; van Klink et al., 2022), which play key roles in ecosystem functioning, and display a diverse range of derived foraging adaptations.

Butterflies are excellent, but undeveloped system to explore animal movement and plant‐insect interactions using camera traps. Butterflies are important pollinators, and their body size is large enough that they may reliably trigger a motion sensor. In this study we assess the use of camera traps to track very specific and small‐scale interactions focused on the derived foraging behaviour of *Heliconius* butterflies. Tropical butterflies of the genus *Heliconius* rely on floral resources, not only as a source of nectar but also as a source of pollen, which, uniquely for butterflies, they actively collect and digest (Gilbert, 1972). Adult *Heliconius* can spend long periods handling a single flower when collecting pollen, building up a pollen load on their proboscis, which is then mixed with saliva and externally digested to release amino acids that are subsequently drawn up the proboscis (Gilbert, 1972). This dietary innovation provides butterflies with an adult supply of amino acids, permitting a prolonged reproductive lifespan, specialised behaviours, and morphological and neuroanatomical changes (Young & Montgomery, 2020).

*Heliconius* have strong site fidelity (Moura et al., 2022) for individual home ranges, which can be less than 200 m in their longest dimension (Murawski & Gilbert, 1986), based largely around a network of pollen plants (Gilbert, 1975). Within these home ranges, *Heliconius* are reported to establish ‘traplines’; spatially and temporally faithful foraging routes that are utilised for long periods. *Heliconius* were first suggested to form traplines following a two‐year study of *H. ethilla* in Trinidad, where observed butterflies patrolled the same flowers regularly to gather pollen, but not in a particular sequence route (Ehrlich & Gilbert, 1973). *Heliconius* butterflies have since been repeatedly reported to forage regularly on the same specific plants, suggesting a sophisticated capacity for spatial navigation (Ehrlich & Gilbert, 1973; Gilbert, 1975; Mallet, 1986). The ability of *Heliconius* to learn the location of resources in their environment is likely linked to their derived brain morphology, and especially the enlargement of the mushroom bodies, insect learning and memory centers (Zars, 2000). In *Heliconius*, these structures are three to four times larger than other Lepidoptera, relative to the size of the rest of the brain, including closely related Heliconiini, such as *D. iulia* and *Agraulis vanillae* (Couto et al., 2023), which occupy similar habitats and overlap in other ecological traits such as host plant preference. *Heliconius* mushroom bodies also show evidence of visual specialisation, and indeed *Heliconius* are able to learn spatial information (Moura, Young, et al., 2023a), and show increased precision in visual discrimination and learning tasks and evidence of enhanced long‐term visual memory retention relative other non‐*Heliconius* butterflies (Couto et al., 2023; Young et al., 2024).

Although spatial learning has been demonstrated in *Heliconius* in insectary conditions (Moura, Young, et al., 2023a), few studies have experimentally explored the ability of *Heliconius* to learn spatial information in natural settings, or evaluated the benefits and origin of this behaviour through comparative studies with other non‐pollen feeding Heliconiini (Gilbert, 1975; Young & Montgomery, 2020). One reason for the lack of comparative and quantitative data is the difficulty of following these butterflies in wild and the low success of mark‐recapture studies – less than 50% success rate for a single recapture (Mallet & Barton, 1989; Moura et al., 2022). As obtaining foraging data in the wild is challenging, here we explore the use of motion‐activated cameras for monitoring butterfly foraging behaviour. Our aim is to test the feasibility of using camera traps to study spatial foraging and to potentially detect evidence of spatially faithful foraging patterns. Using motion‐sensitive cameras, we recorded data on floral visitation in time and space using marked individuals of three pollen‐feeding species of *Heliconius* (*H. erato*, *H. melpomene* and *H. sara*), and two closely related, non‐pollen feeding Heliconiini species (*Dryas iulia* and *Dryadula phaetusa*) in a large outdoor insectary. We use these data to explore key questions regarding the foraging strategy of Heliconini genera, the effects of sex and species on their individual foraging behaviours, and their temporal and spatial foraging patterns.

## 2 MATERIALS AND METHODS

### 2.1 Butterfly rearing

**FIGURE 1:**
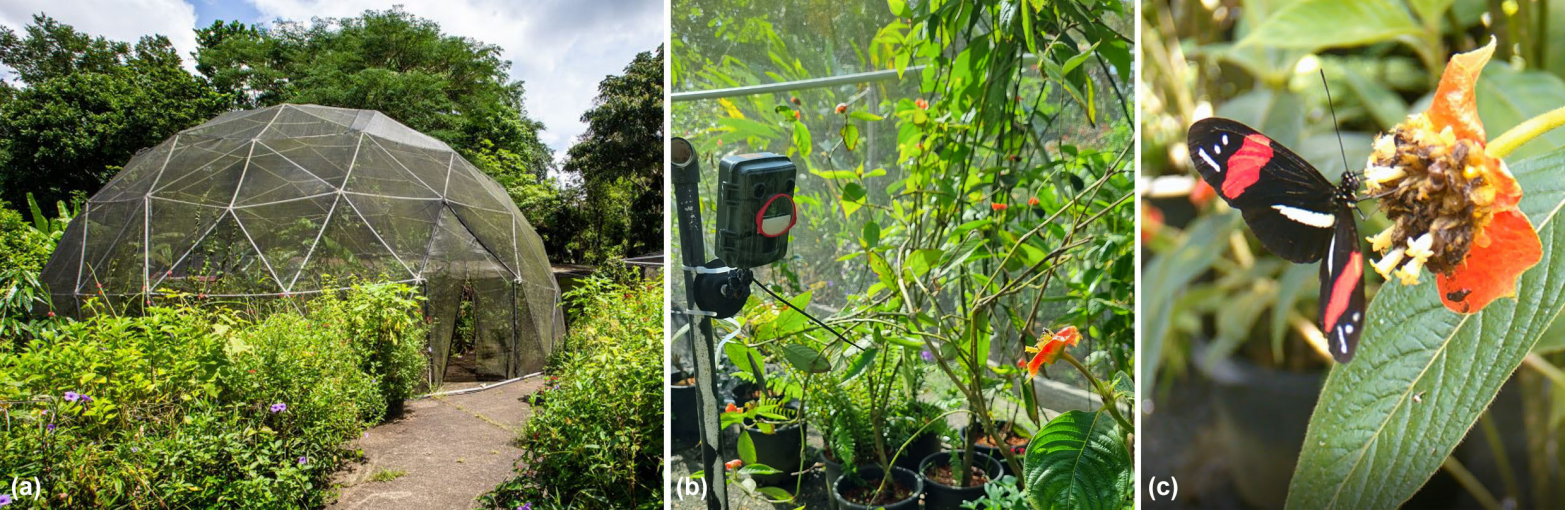
(a) Outdoor dome where the experiments were performed. (b) Camera trap with extra lens located 30 cm of a *Palicourea* flower. (c) Screenshot from a video recording showing a marked *Heliconius erato* feeding from a *Palicourea* flower.

Stock populations of *Heliconius erato demophoon*, *H. melpomene rosina*, *H. sara*, *D. iulia* and *D. phaetusa* were established in outdoor insectaries at the Smithsonian Tropical Research Institute, Gamboa, Panama, from natural populations in the surrounding area. Eggs and larvae were raised in their respective hostplants (*H. erato*, *D. iulia* and *D. phaetusa* on *Passiflora biflora*, *H. melpomene* on *P. menispermifolia* and *H. sara* on *P. auriculata*). Stock populations of freshly eclosed adults were kept in standardised conditions (2 m^3^ outdoor cages with natural sunlight) and fed with natural flowers (*Stachytarpheta* sp., *Pentas* sp. and *Palicourea tomentosa*) prior to their inclusion in the experiments. Each butterfly was individually marked with a silver marker pen (Figure 1c), and their sex recorded.

### 2.2 Experimental cage

The experimental cage consisted of a circular dome of approximately 12 m in diameter and 10 m in height covered with black mesh and located outdoors (Figure 1a). Inside the dome, motion‐activated cameras (Mini Wildlife Trail Camera Version 18,022, K&F Concept ©, Shenzhen, China) were positioned 30 cm away from a floral resource to be able to detect the small body of a butterfly (Figure 1b). To permit adequate focus at close proximity, extra external lens (half frame of +3.00 glasses, Figure 1b and Figure S1) were added in front of the camera lenses, an adjustment which provided a clear image of a marked butterfly (Figure 1c and Video 1). Cameras were set to have a 0.2 s trigger time, maximum motion sensitivity, and to record 20 s videos between 8 AM and 4 PM, the period of highest activity for *Heliconius*.

**VIDEO 1:** Example of video taken by one of the motion‐activated cameras. It contains temperature, date and time information. Species in the video: *Heliconius sara* and *Heliconius erato* on a *Palicourea* flower.

**FIGURE 2:**
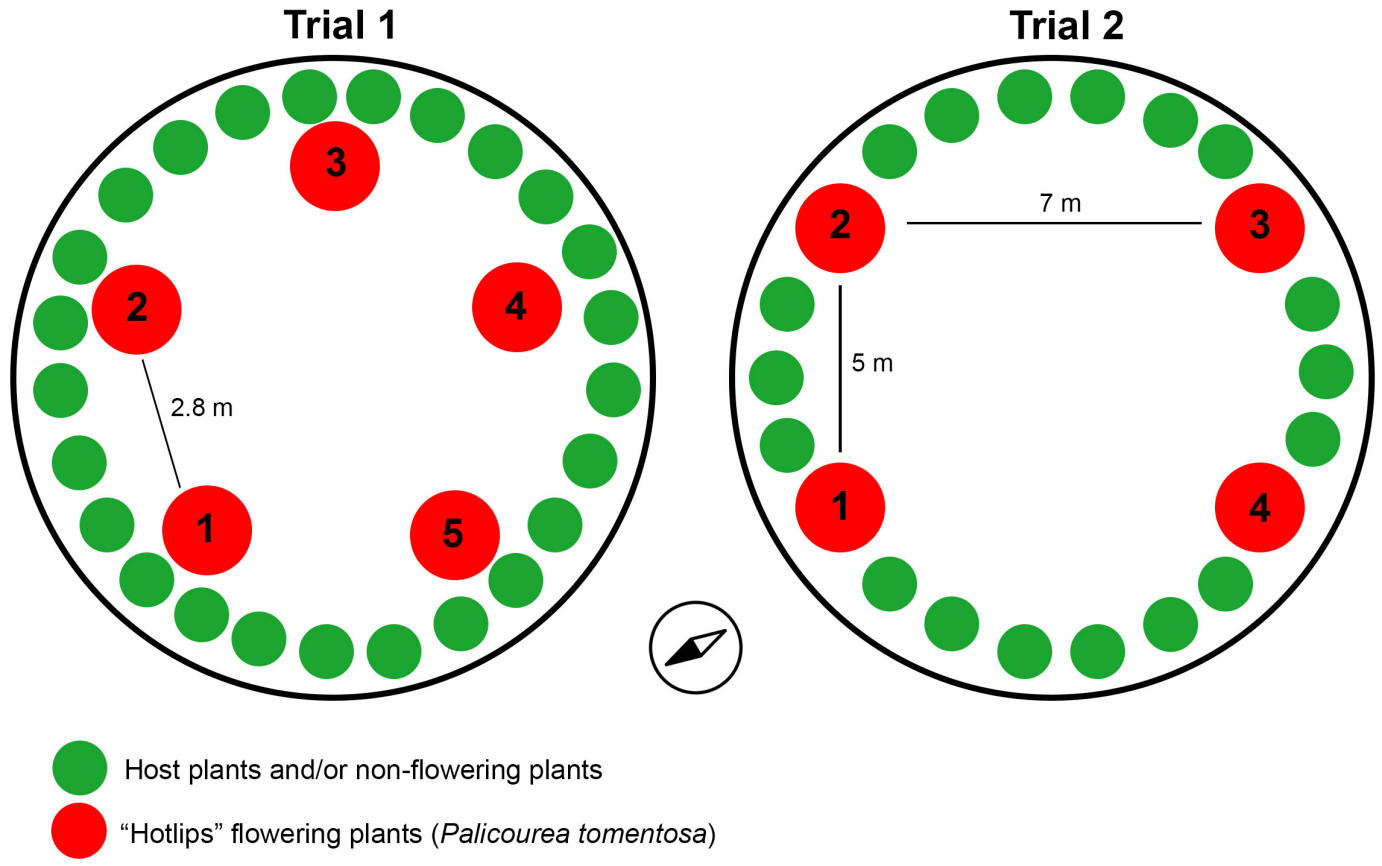
Schematic figure of plant distribution inside the outdoor dome during trials 1 and 2. Red wider circles with numbers are flower clusters and green smaller circles are non‐flowering plants. Black arrow of the compass pointing north.

Each camera was pointed towards one flower in each plant cluster, created by tightly clumping a group of potted plants inside the dome using *Palicourea tomentosa* (synonyms: *Psychotria poeppigiana* and *Cephaelis tomentosa*), which has terminal inflorescences that are capitate with red bracts and yellow flowers (Figures 1c and 2). The pollen of this flower is documented to be highly consumed by *Heliconius* (Estrada & Jiggins, 2002) and visited by other Heliconinii species that exploit it as a nectar resource (*personal observations*). This permitted comparable data collection across both pollen and non‐pollen feeding species. *Palicourea tomentosa* blooms are stable and produce new flowers each day at the same spot providing a temporally reliable resource (Coelho & Barbosa, 2004; Valois‐Cuesta et al., 2009). During the experiment, a few terminal inflorescences stopped blooming, and the camera position was therefore adjusted to capture another flower in the same cluster. Plant clusters were created to space out the cameras around the dome (Figure 2).

### 2.3 Experimental procedure

At the beginning of each experiment adults were moved to the experimental cage, where butterflies could fly freely, with access to floral and hostplant resources in semi‐natural conditions. We performed two experimental trials: (i) In the first trial, performed from November to December of 2022, five plant clusters (with ~5 available flowers) were created inside the dome in a pentagon shape, 2.8 m between the clusters, each with one camera trap adjacent to a flower (Figure 2). In total, 15 days of data were collected with 58 individuals released inside the dome (16 *H. erato*, 16 *H. melpomene*, 14 *H. sara* and 12 *D. iulia*). (ii) In the second trial, we aimed to reduce competition that was observed during the first trial by arranging fewer flower clusters more distantly from one another, with four plant clusters organised in a rectangle, with 5 to 7 m between clusters (Figure 2). In this trial, butterflies were divided into five groups of two species (total of 19 *H. erato*, 13 *H. melpomene*, 11 *D. iulia* and 11 *D. phaetusa*), always pairing one *Heliconius* species with a non‐*Heliconius* species with the same number of individuals. Each group was held in the dome for 10 days, and cameras were active during the last 5 days. Groups were run consecutively between March and May of 2023.

### 2.4 Video and data analysis

During the trials, each feeding event detection by the camera trap produced one video. These videos were reviewed and annotated using the image analysis software *Timelapse 2* (Greenberg et al., 2019). For each video, the individual identity and species of the butterfly present were recorded. The videos also contained information about date, time of the day and temperature. Therefore, datasets of spatial and temporal foraging patterns were obtained by counting video appearances and grouping visits for each individual by date, time and flower cluster (Figure 2). Individuals with less than two recorded visits were removed from the dataset. Activity pattern graphs were made using *density* in ‘ggplot2’ package (Wickham, 2016) in R (R Core Team, 2023), and consist of a representation of the distribution (using kernel density estimate, a smoothed version of a histogram) of videos recordings for each species and/or sex during time.

Differences in visitation rates/patterns between sex and species were calculated using linear mixed‐effect models (‘lmer’) implemented with the ‘lme4’ package in R (Bates et al., 2015) using a *poisson* distribution for raw data or *binomial* distribution for proportion data. We included “individual” as random factor when response variable was per individual and “week” as random factor for trial 2 (as stated above, trial 2 was divided in 5 groups, each on a different week), followed by analysis of deviance (ANOVA Type II) and Tukey's post hoc tests with the packages ‘car’ and ‘emmeans’ in R (Fox & Weisberg, 2019; Lenth, 2023). In addition, Chi‐squared tests (R built‐in package ‘stats’) were used to compare the absolute number of visits observed to each flower against the expected number assuming random foraging (presumed random probability of 0.2 for trial 1 and 0.25 for trial 2 for each flower) for each individual. Individuals with less then 3 flower visits were removed from the analysis.

Finally, the level of sequence repetition in flower visits was explored to assess whether there was any evidence of potential trap line foraging in this experimental set up. Using individual visit sequence data, we used determinism analysis, a statistical metric to quantify the predictability of sequential behaviours (Ayers et al., 2015). The denominator of the determinism (DET) varies between 0, indicating that the individual never repeats the same sequence, and 1, indicating that the individual always repeats the same sequence. For each individual the DET was calculated using the minimal sequence length of recurrent visits of 3 different flowers (Ayers et al., 2015).

## 3 RESULTS

### 3.1 Camera traps capture natural, individual activity patterns

In the first trial, 1512 videos detected butterflies feeding from flowers over a period of 120 hours. Of the 58 individually marked butterflies, 51 (87.9%) were caught in the videos (Table 1 and Table S1). *H. sara* was the species most frequently recorded on the cameras (*n* = 718), followed by *H. erato* (*n* = 331), *D. iulia* (*n* = 283) and *H. melpomene* (*n* = 180). In the second trial, a total of 330 videos captured feeding events, from a total of 200 h. Of the 54 marked butterflies, divided in 5 groups, 35 (64%) were detected in the videos (Table 1 and Table S1). The number of recordings varied depending on the week, but overall *H. erato* had the greatest number of recordings (*n* = 166), followed by *D. phaetusa* (*n* = 92), *H. melpomene* (*n* = 45) and *D. iulia* (*n* = 27).

**TABLE 1:** Number of marked individuals released in the dome, number of individuals detected by the camera traps, percentage of detected individuals and number of videos for each species in each trial.

Species	Marked	Detected	%	No. of videos
1st trial				
*Heliconius erato*	16	16	100	331
*Heliconius melpomene*	16	12	75	180
*Heliconius sara*	14	12	85.7	718
*Dryas iulia*	12	11	91.6	283
Total	58	51	87.9	1512
2nd trial				
*Heliconius erato*	19	15	78.9	166
*Heliconius melpomene*	13	9	69.2	45
*Dryadula phaetusa*	11	5	45.4	92
*Dryas iulia*	11	6	54.5	27
Total	54	35	64.8	330

**FIGURE 3:**
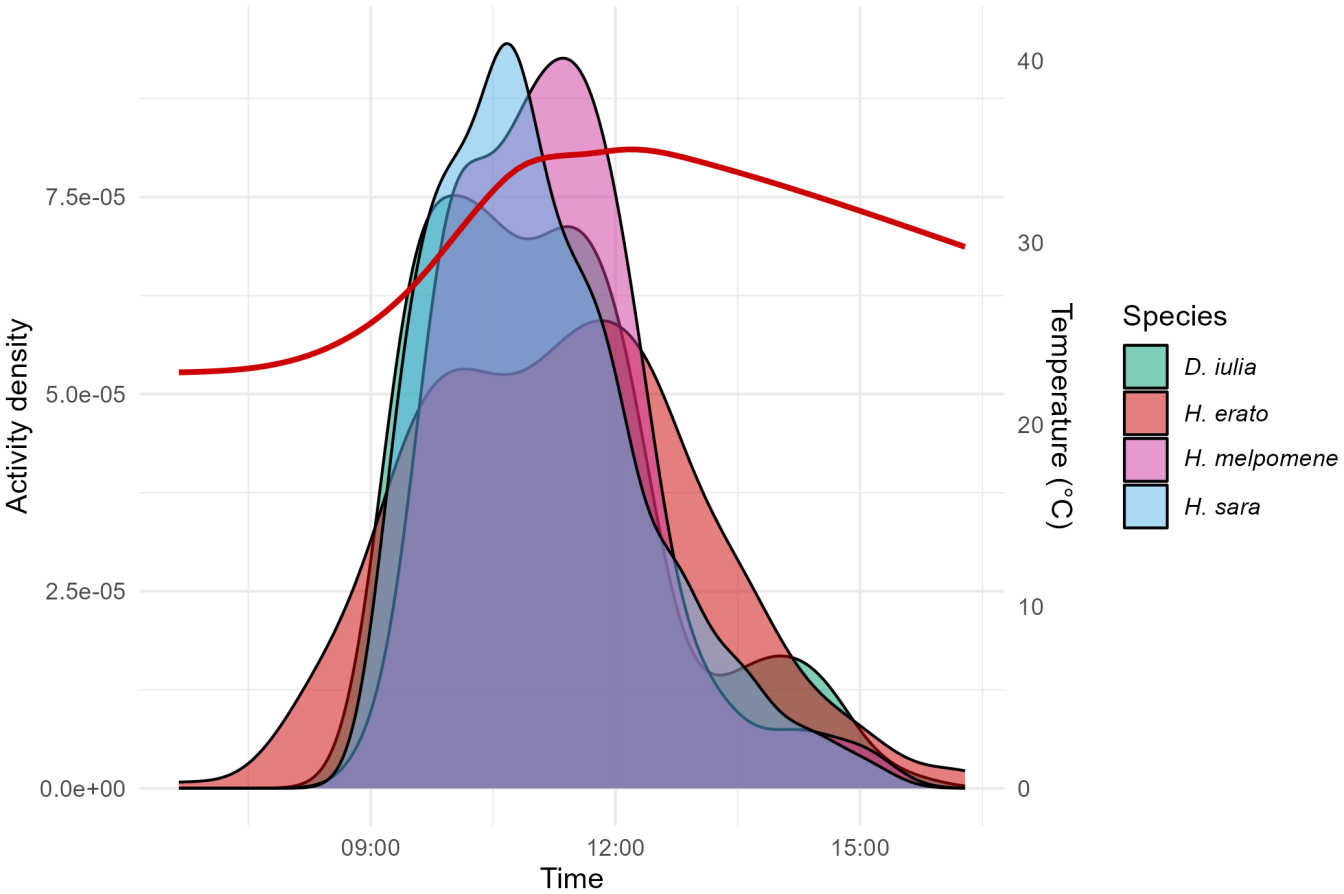
Activity pattern and temperature (°C) from camera‐trap data for the first trial. Kernel density functions were used to depict butterfly activity sampled via camera trapping. Red line, mean temperature during the day.

In both trials, species activity was higher in the morning. In trial 1, it was higher between 09:30 and 12:00 for all species (Figure 3). Temporal niche partitioning was not observed since species' coefficient of overlap was very high between all species (*D. iulia* vs. *H. erato* = 0.81, *D. iulia* vs. *H. melpomene* = 0.87, *D. iulia* vs. *H. sara* = 0.90, *H. erato* vs. *H. melpomene* = 0.76, *H. erato* vs. *H. sara* = 0.77, *H. melpomene* vs. *H. sara* = 0.88; Figure 3). Although activity time variance was significantly different ($X_{3}^{2}$ = 9.1, *p* = .027), post‐hoc tests show this was driven by a difference between *H. erato* and *H. sara* (post‐hoc: *t* = 2.8, *p* = .01; *H. erato* mean = 11 h 18 min AM, se ± 4 min; *H. sara* mean = 11 h 03 min AM, se ± 3 min).

**FIGURE 4:**
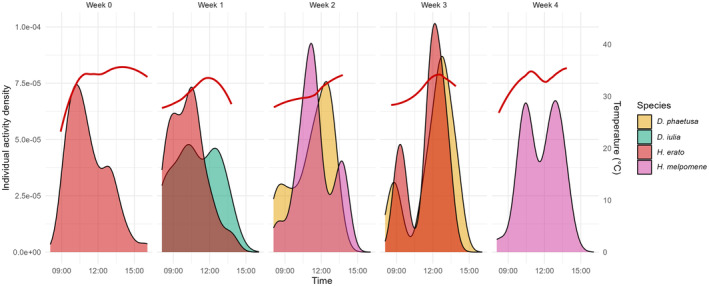
Activity pattern and temperature (°C) from camera‐trap data for the second trial in each week. Kernel density functions were used to depict butterfly activity sampled via camera trapping. Red line, mean temperature during the day.

In trial 2, where only two species were present at the same time, niche partitioning was also not observed (Figure 4), as the coefficient of overlap was also high: in week 1 (*D. iulia* and *H. erato*) = 0.72, week 2 (*H. melpomene* and *D. phaetusa*) = 0.62, or week 3 (*H. erato* and *D. phaetusa*) = 0.82. In week 0, only *H. erato* was released in the dome, and in week 4, *H. melpomene* and *D. iulia* were released but *D. iulia* was registered only once. Activity time was not significantly different between species ($X_{3}^{2}$ = 3, *p* = .261).

**FIGURE 5:**
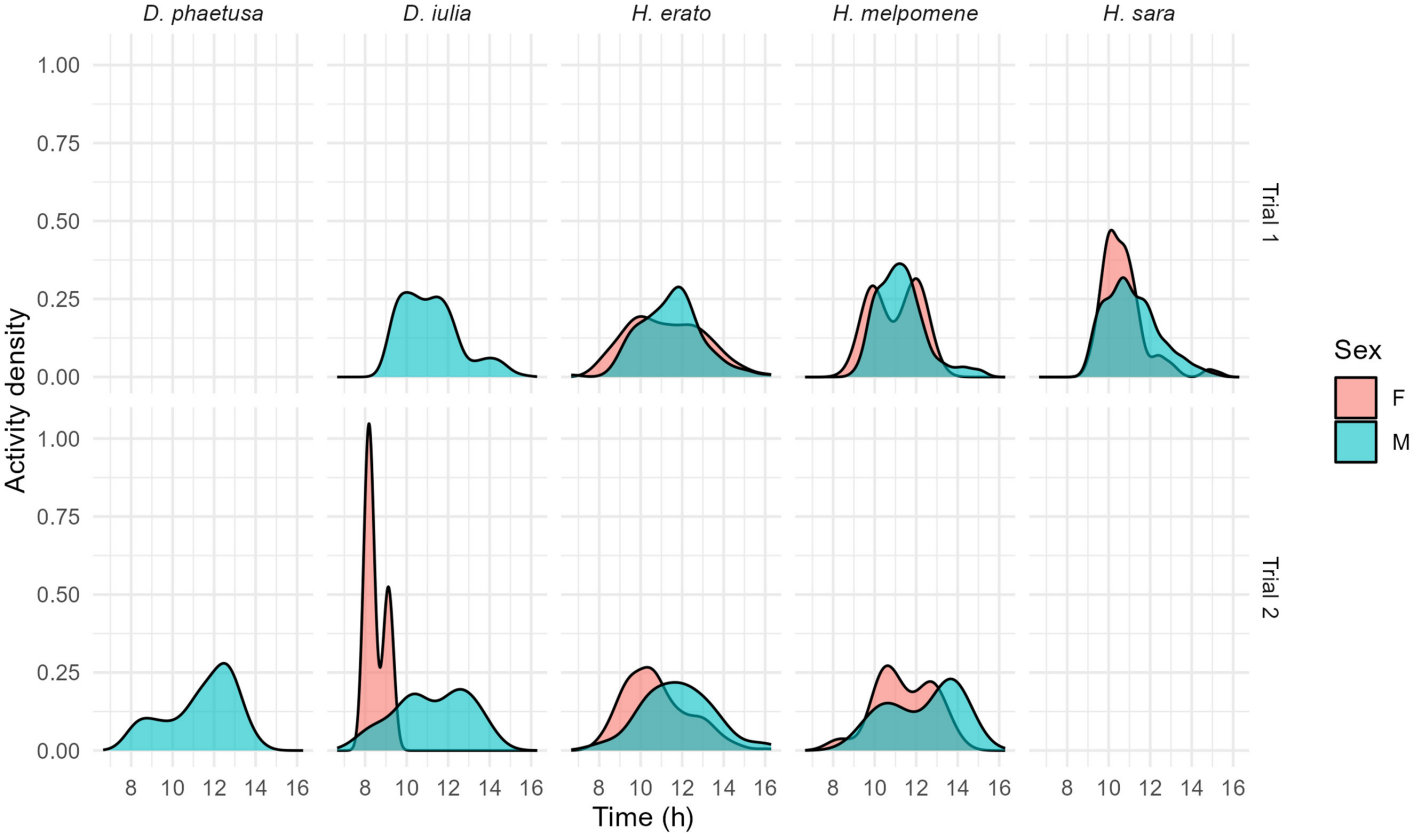
Activity pattern from camera trap data divided by species and sex during the first and second trial.

In general, females were active earlier than males (Figure 5). During trial 1, female and male activity was significantly different (sex, $X_{1}^{2}$ = 11.6, *p* < .001; species, $X_{3}^{2}$ = 17.2, *p* < .001; sex:species, $X_{3}^{2}$ = 1.27, *p* = .735). In trial 2, the difference between female and male activity was more prominent (sex, $X_{1}^{2}$ = 17.1, *p* < .001; species, $X_{3}^{2}$ = 14.2, *p* = .002; sex:species, $X_{3}^{2}$ = 4.1, *p* = .249).

The first trial was performed at the end of the rainy season and the temperature observed during their activity time reached a maximum of 41°C around 12:00 and minimum of 19°C early morning, with a mean of 32.5°C (Figure 3). The second trial was performed at the end of the dry season and the temperature during their activity time reached a maximum of 38°C around 1 PM and a minimum of 24°C in the morning, with mean of 31.7°C (Figure 4). Foraging activity was not influenced by temperature (trial 1, *t* = 1.86, *p* = .065; trial 2, *t* = 0.52, *p* = .602).

### 3.2 Heliconiini species vary in their spatial foraging patterns

The number of recordings varied between the flower clusters. In the first trial, the most visited floral site was cluster 3, located Southeast, with especially high visitation rates by *H. sara* (Figure S2). The least visited was cluster 4, which was in the most shaded part of the dome, and where most of the nocturnal roosts (sleeping sites) were observed (*personal observation*). During the second trial, although the position was slightly different from trial one, cluster 3 was again the most visited floral station (Figure S2). In trial 1, the most preferred floral station varied with time (Date, $X_{1}^{2}$ = 14.09, *p* < .001; Camera, $X_{4}^{2}$ = 21, *p* < .001). This was not the case for trial 2 (Date, $X_{1}^{2}$ = 3.19, *p* = .073; Camera, $X_{3}^{2}$ = 3.43, *p* = .329).

In trial 1, species differed in foraging patterns, with some individuals visiting more flowers per day than others (Species, $X_{3}^{2}$ = 13.6, *p* = .003; Sex, $X_{1}^{2}$ = 0.1, *p* = .74). Individuals of *D. iulia* visited more flowers per day than other species (post‐hoc *Tukey*, *H. erato*, *t* = 2.5, *p* = .058; *H. melpomene*, *t* = 2.9, *p* = .021; *H. sara*, *t* = 3.5, *p* = .005). In trial 2, where competition was reduced, the average number of flowers used per day did not differ between species (Species, $X_{3}^{2}$ = 3.7, *p* = .29; Sex, $X_{1}^{2}$ = 0.0008, *p* = .97), but less data was available for non‐*Heliconius* genera. Comparing the proportion of flower stations visited per individual throughout the experiment using only the three species presented at both trials (*H. erato*, *H. melpomene and D. iulia*), no significant differences were found between trial 1 and 2 (Trial, $X_{1}^{2}$ = 1.44, *p* = .22; Species, $X_{2}^{2}$ = 3.9, *p* = .14; Trial:Species, $X_{2}^{2}$ = 1.39, *p* = .49), suggesting that butterflies effectively explored the dome in a similar way during both trials despite the interspecific competition.

To test whether individuals were foraging randomly, we compared the pattern of visits to each individual flower cluster against a null expected of visitation rates based on a random distribution across all floral clusters. This revealed that across trial 1, most individuals showed non‐random foraging patterns. We also observed significant differences in the tendency to forage randomly between species (Species, $X_{3}^{2}$ = 11.7, *p* = .008; Sex, $X_{1}^{2}$ = 3.2, *p* = .071, post‐hoc: all comparisons *p* > .05), with *D. iulia* (25%) most often presenting foraging pattern most consistent with the null expectation (Table S2). In contrast, in trial 2, most individuals showed random foraging patterns and there was no difference between species or sex (Species, $X_{3}^{2}$ = 4.3, *p* = .223; Sex, $X_{1}^{2}$ = 2.5, *p* = .107) (Table S2). This result suggests that the reduced number of individuals in the dome during the second trial influenced foraging patterns butterflies.

Although most individuals showed non‐random foraging patterns, we found no evidence of route repeatability in the dataset using DET. We found very low repeatability estimates for all species in the first trial (mean *D. iulia* = 0.11, *H. erato* = 0.06, *H. melpomene* = 0.006, *H. sara* = 0.07) and in the second trial (mean: *H. erato* = 0, *D. iulia* = 0, *H. melpomene* = 0 and *D. phaetusa* = 0.17). However, we note that this result is likely confounded by the low number of floral clusters visited by each individual. For *Heliconius* in particular, in trial 1, most individuals were faithful to one cluster, negating any possibility of consistent foraging sequences.

### 3.3 Butterflies constantly returned to the same flower

**FIGURE 6:**
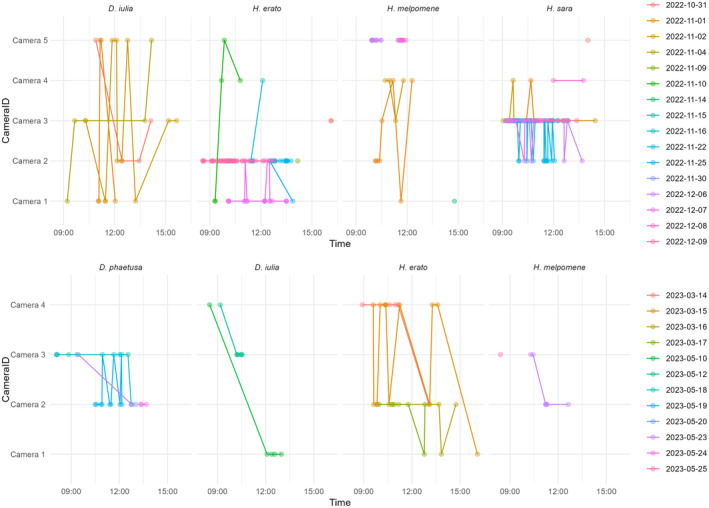
Examples of individuals foraging pattern in trial 1 (top) and trial 2 (bottom) in space and time. Each dot is a visit to a flower/camera and lines connect the visits on the same day. Multiple consecutive dots in the same date and camera represents feeding bouts. Colour indicates date. For example: *Drya iulia* (top left) visits many different flowers across the day while *Heliconius sara* (top right) repeatedly visits the same flower.

We observed that feeding was grouped in short periods of intense visits to the same flower, which we refer to as feeding bouts (Figure 6). During these feeding bouts, individuals would repeatedly return to the same flower multiple times within a short period of time. The maximum time between feeding intervals to be included in the same bout was stipulated to be 20 min, although the average interval was 7.8 min in trial 1 and 9.5 min in trail 2. The characteristics of these feeding bouts varied between species.

In the first trial, total duration of feeding bouts varied between species, and was shortest for *D. iulia* (mean = 26.4 min) and longest for *H. sara* (mean = 78.6 min) (Species, $X_{3}^{2}$ = 13.4, *p* = .003; Sex, $X_{1}^{2}$ = 1.97, *p* = .15, post‐hoc: *D. iulia* – *H. sara*, *t* = −3.37, *p* = .03). The number of visits during a feeding bout also varied between species (Species, $X_{3}^{2}$ = 10.14, *p* = .017; Sex, $X_{1}^{2}$ = 0.03, *p* = .84), with *H. sara* having an average of 11.6 visits per bout, followed by *D. iulia* 7.2 visits, *H. erato* 5.9 visits and *H. melpomene* 5.5 visits. There was also variation in the duration of intervals between the visits (Species, $X_{3}^{2}$ = 15.36, *p* = .001; Sex, $X_{1}^{2}$ = 4.52, *p* = .033), with *D. iulia* having shorter intervals (mean = 3.6 min) than either *Heliconius* (post‐hoc: *D. iulia* – *H. erato t* = −3.37, *p* = .011; *D. iulia* – *H. melpomene*, *t* = −2.9, *p* = .031). In the second trial, we found no difference in the duration of feeding bouts (Species, $X_{3}^{2}$ = 0.24, *p* = .96; Sex, $X_{1}^{2}$ = 0.4, *p* = .52), number of visits (Species, $X_{3}^{2}$ = 0.17, *p* = .98; Sex, $X_{1}^{2}$ = 1.04, *p* = .31) or visit intervals (Species, $X_{3}^{2}$ = 1.45, *p* = .69; Sex, $X_{1}^{2}$ = 0.11, *p* = .74).

**FIGURE 7:**
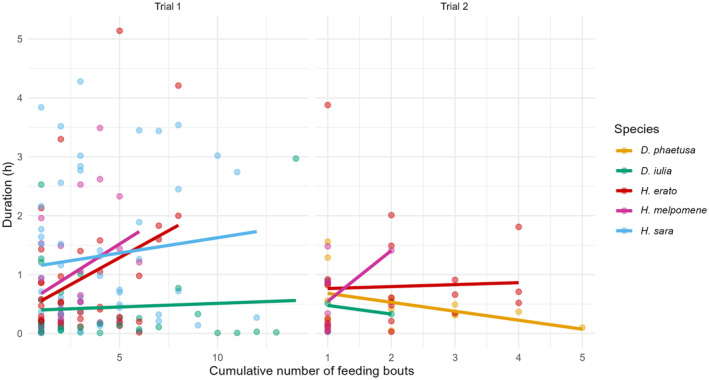
Cumulative number of feeding bouts on feeding bout duration (hours) in each trial. Each dot is an individual feeding bout.

As time progressed, the feeding bouts lasted longer on average (Figure 7). In the trial 1, the duration of feeding bouts was predicted by the individual's species, and their number of feeding bouts (Feeding bout, $X_{1}^{2}$ = 5.7, *p* = .016; Species, $X_{3}^{2}$ = 10.4, *p* = .015; Sex, $X_{1}^{2}$ = 1.7, *p* = .18), with a very weak effect of number of feeding bouts on *D. iulia* feeding duration (correlation = 0.06) compared to other species (*H. erato* = 0.34, *H. melpomene* = 0.33, *H. sara* = 0.12). In trial 2, the number of feeding bouts also affected the duration of bouts in a similar direction, although not significantly again likely reflecting the lower sample size (Feeding bout, $X_{1}^{2}$ = 0.16, *p* = .68; Species, $X_{3}^{2}$ = 0.34, *p* = .95; Sex, $X_{1}^{2}$ = 0.48, *p* = .48). The number of feeding bouts had a negative effect on *D. iulia* = −0.20 and *D. phaetusa* = −0.43, positive on *H. erato* = 0.04 and *H. melpomene* = 0.51 (Figure 7).

## 4 DISCUSSION

In this study we explored the potential application of camera traps in the study of insect foraging behaviour, with a focus on Heliconiini butterflies, which show intergeneric differences in floral resource use, longevity, and foraging. We demonstrate that camera traps capture individual flower visitation over multiple times and locations and use our initial experiments to describe some features of their spatial and temporal foraging patterns. Below we discuss possible factors affecting those patterns, and directions for future use of camera traps in monitoring butterfly behaviour.

Our data effectively capture species‐level effects of time and location on foraging behaviour. While previous studies of unmarked insects have reported no significant differences between camera trap and humans at detecting Lepidoptera (Naqvi et al., 2022), we expect that some butterfly activity might have been undetected by the motion camera, but the reflection of behavioural trends observed in field studies in our data suggest this does not strongly impact our ability to infer patterns of behaviour. We attempted to quantify this effect by carrying out a series of short observations during trial 2 (totalling 10 h) but during these periods we observed only 8 feeding attempts (0.8 feeding bouts per hour compared to 1.65/h as recorded by the camera traps), suggesting the presence of an observer impacted butterfly foraging. However, of these 8 attempts all were recorded in the camera traps. Nevertheless, we did observe some limitations with the camera traps themselves, such as some cameras being triggered by the movement of flowers caused by rain and wind. In addition, while we had no difficulty in identifying species and single individuals because of the specific marking on their wings, we believe that this task would be more difficult in the wild with unmarked individuals.

As reported in human‐recorded field and insectary studies (Hebberecht et al., 2023), Heliconiini butterflies showed higher activity in the morning with a strong temporal niche overlap. *Heliconius erato* was previously observed to feed from *P. tomentosa* at the same time window, between 9 AM and 1 PM, likely driven by higher nectar concentration in the flowers (Valois‐Cuesta et al., 2009). Butterflies might adjust their temporal activity based on ecological traits such as solar position and flower opening. Our observed decrease in activity in the afternoon, despite temperatures being still suitable for foraging, reinforce the inference of strong temporal niche conservation in foraging behaviour. Moreover, activity may have also been lower in the afternoon due to heavy rains in this period (*personal observation*). Similar niche overlap was also observed in previous butterfly research, which included some Heliconinii butterflies (Riva et al., 2023). Daily activity patterns of Mediterranean butterflies on *Lavanda latifolia* shrubs showed butterflies were also more dominant between 10 AM and 1 PM even if the maximum flower reward was early morning and late afternoon (Herrera, 1990).

We observed differences in foraging activity between males and females, with females initiating foraging earlier in the morning on average. Again, this mirrors observations from field studies of *Heliconius* (Murawski & Gilbert, 1986). This is hypothesised to reflect the impact of resource allocation differences, as females have a greater need for pollen‐derived amino acids in their diet to maintain egg production (Gilbert, 1972). According to Murawski and Gilbert (1986), females forage earlier due to competition, enabling larger pollen loads to be collected, with later periods of activity concentrated on host plant foraging. In contrast, males, having a less immediate pollen demand, would forage more widely, combining activities of foraging with searching for receptive females (Murawski & Gilbert, 1986). Our results, however, demonstrate that non‐pollen feeding Heliconiini may share this sex‐difference in activity, suggesting that nectar might also be a sufficiently valuable resource for females to favour shifts in daily activity patterns to facilitate feeding prior to searching for hostplants.

An interesting feature of Heliconiini foraging, which we believe is previously undescribed at this level of detail, is the repetition of floral visits in short periods of time, which we refer to as foraging bouts. These foraging bouts could be interpreted as a defence mechanism, in which butterflies would “defend” their flowers against “invaders” during foraging. However, given individuals often shifted their favourite flower cluster over time, and the observation that these bouts seemingly occur in the absence of intra‐individual interactions, we consider this unlikely. An alternative hypothesis is that this behaviour could be related to visual cue capture for spatial foraging. By repeatedly revisiting the same resource over a short time frame, an individual increases the capture of visual information around a positively rewarding resource, potentially aiding the formation of spatial memory in a manner akin to orientation flights in Hymenoptera (Capaldi & Dyer, 1999; Degen et al., 2016; Zeil et al., 1996). The longer duration of these feeding bouts in *Heliconius*, and the greater tendency of feeding experience to reduce searching behaviour for new flowers, may hint at an increased importance of this learning in pollen‐feeding species.

In addition to capturing temporal variation in floral visitation rates, our data also captures information about spatial foraging. Some flower stations were more explored than others, not only across all individuals in a trial, but also at an individual level. Preference for southerly floral resources likely reflects attraction towards high‐light conditions, but could also reflect the position of the most rewarding flower. These effects were weaker in trial 2, which may have been due to lower statistical power, but it is also possible that differences observed in spatial foraging patterns could be explained by the presence of other butterflies foraging simultaneously, which was reduced in trial 2. For example, bumblebees avoid visiting plants that received frequent visits by others (Makino & Sakai, 2004), which might may also be the case for butterflies, as they avoided flowers that were occupied by conspecifics (Moura, Cardoso, & Montgomery, 2023b). Heliconiini butterflies feeding on nectar and pollen, a limited resource inside the flower, might have to change their pattern during the day to avoid flowers that have their pollen/nectar removed. To test this, we would need further analyses on the amount of pollen and nectar produced and collected in *Palicourea* flowers. In addition, although inflorescences of *Palicourea* last for many days, inflorescences will wither after 10–15 days favouring foraging strategies that are sufficiently flexible to incorporate new resources. As such, occasionally sampling other plants is advantageous in the wild where plants change in value throughout time. This may suggest that even in *Heliconius* foraging behaviour may be more flexible and interspaced with random sampling than in traplining bees, which have a strong preference for direction and time saving (Lihoreau et al., 2011). The differences in foraging behaviour might be due to the differences in ecology, but require further investigation.

Traplining, by definition, is the feeding strategy in which an individual visits food sources on a regular, repeatable sequence (Janzen, 1971). Most research on trapline following behaviours has been done in bees (Kembro et al., 2019; Klein et al., 2017; Makino & Sakai, 2004; Ribbands, 1949) and hummingbirds (Tello‐Ramos et al., 2022), where both rely on floral resources and to support energetically costly flight behaviour. In our experiments we did not observe repeatable sequences in foraging behaviour, but the tendency for individuals to use a small number of floral clusters likely negates this possibility. However, such behaviour does provide evidence of consistent spatial foraging. Indeed, one of the benefits of having a trapline is to reduce the time spent searching for new food plants each day, so if an individual has a small home range with sufficient food resources, forming a trapline might not be necessary. In a long‐term study about pollen flow between *Psiguria* flowers, marked *Heliconius* individuals regularly visited the same flowers and host plants in a relative small home range, not more than 200 m^2^ (Murawski & Gilbert, 1986). *Psiguria* flowers are a temporally constant resource, and once butterflies find them, they keep using this resource even if the size of the flowers decrease, suggesting a strong learning behaviour and space memory (Dixit et al., 2020). In our experiment, butterflies repeatedly fed from the same group of flowers, potentially a result of the artificially small environment we created inside the dome, and the high density of floral resources within a cluster. In trial 1 this effect was stronger in *Heliconius* than in *Dryas*, again suggesting a potential refinement of spatial fidelity during the evolution of pollen‐feeding. These past studies, and our own, therefore suggest that resource fidelity and trap‐lining in *Heliconius* is likely shaped in response to the distribution, quality and stability of the available resources.

## 5 CONCLUSION

*Heliconius* and other Heliconiini have many ecological similarities, such as the use of *Passiflora* hostplants and the same flower resources. The performance of foraging behaviour can be expected to be similar. However, *Heliconius*, which have expanded mushroom bodies, the key site of insect learning and memory, have improved performance in visual cognitive tasks relevant to foraging in traplines such as visual non‐elemental learning and long‐term memory (Couto et al., 2023; Young et al., 2024; Young & Montgomery, 2023). However, a lack of field data on the foraging behaviour of non‐pollen feeding Heliconiini leaves inferences of what behavioural strategies are derived in *Heliconius*, and associated with pollen feeding, inherently limited. Here, we observed that *Heliconius* are more “loyal” to the flowers, more consistently coming back for the same floral cluster, while *D. iulia* and *D. phaetusa* visited more different flowers per day, with shorter feeding bouts than *Heliconius* species. This may reflect the reduced time investment of nectar feeding, but the fidelity of *Heliconius* to particular feeders across the trial may provide support for the contention that the foraging behaviours of *Heliconius* are derived with respect to their closest relatives, with a greater role of spatial learning and repeated resource visitation (Gilbert, 1972; Murawski & Gilbert, 1986; Young & Montgomery, 2023).

More broadly, we have shown that the use of camera traps is a powerful tool to gather information about foraging behaviour in butterflies. Our data reveal general trends such as temporal pattern of species activity that overlap, but with consistent differences in the daily activities of males and females. Such data can provide important information about natural activity patterns, and potentially provide a context for experimental perturbation studies. Future studies can build upon our results and evaluate the influence of environmental traits such as the quality and quantity of nectar and pollen resources, abiotic conditions, insectivorous predator activity, and butterfly‐butterfly interaction.

## AUTHOR CONTRIBUTIONS

**Denise Dalbosco Dell'Aglio:** Conceptualization (equal); data curation (lead); formal analysis (lead); investigation (lead); methodology (lead); writing – original draft (equal). **Owen W. McMillan:** Funding acquisition (equal); project administration (equal); resources (equal); supervision (equal); writing – review and editing (equal). **Stephen Montgomery:** Conceptualization (lead); funding acquisition (lead); project administration (equal); resources (equal); supervision (equal); writing – original draft (equal); writing – review and editing (equal).

## FUNDING INFORMATION

This research was supported by Natural Environment Research Council (NERC) Independent Research Fellowship (IRF) (NE/N014936/1) and a European Research Council (ERC) Starter Grant (758508) to S.H.M. which supported a Research Associate Postdoc to D.D.D.

## CONFLICT OF INTEREST STATEMENT

The authors have no conflict of interest to declare.

## Supporting information


Data S1.

## Data Availability

The data supporting this study are available in the Dryad repository (https://datadryad.org) under the accession number https://doi.org/10.5061/dryad.gmsbcc2wg.
