# Supplementary material for: Using motion‐detection cameras to monitor foraging behaviour of individual butterflies
Source: Ecol Evol. 2024 Jul 21;14(7):e70032. doi: 10.1002/ece3.70032 (PMC11260874; doi:10.1002/ece3.70032)
Supplement: Supplementary file 1 — Data S1. [file ECE3-14-e70032-s001.docx]

**Supplementary Data**

**Using motion-detection cameras to monitor foraging behaviour of individual butterflies**

**Authors:**

Denise Dalbosco Dell’Aglio¹^,2^, Owen W McMillan¹, Stephen Montgomery¹^,2^

**Affiliations:**

1. Smithsonian Tropical Research Institute, Panama City, Panama

2. School of Biological Science, University of Bristol, Bristol, UK


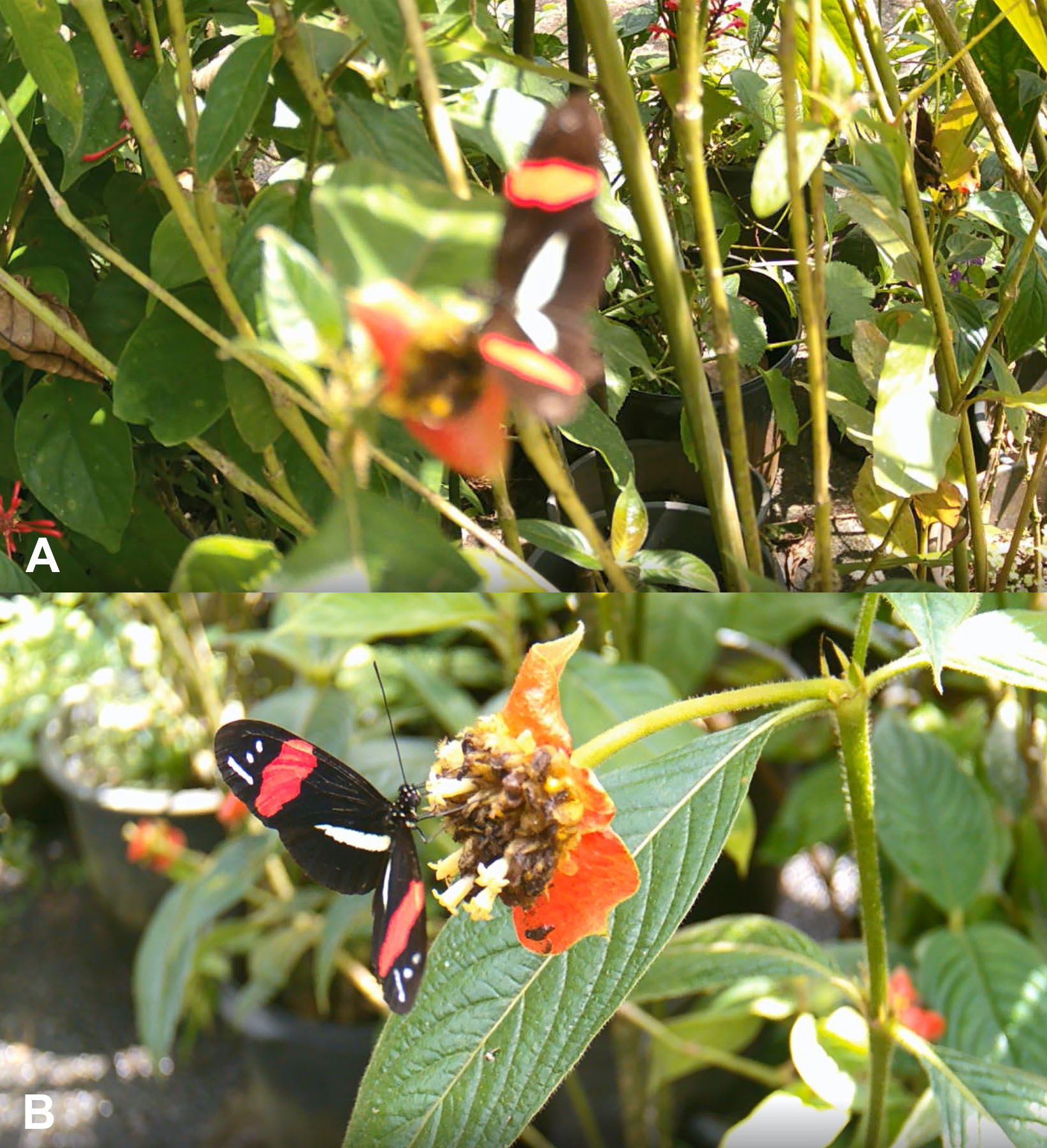


**Supplementary Figure 1.** Screenshot of a video recorded by the camera traps. A. Without the extra lenses. The butterfly is out of focus, difficult to identify. B. With the extra lenses. The focus in on the butterfly.


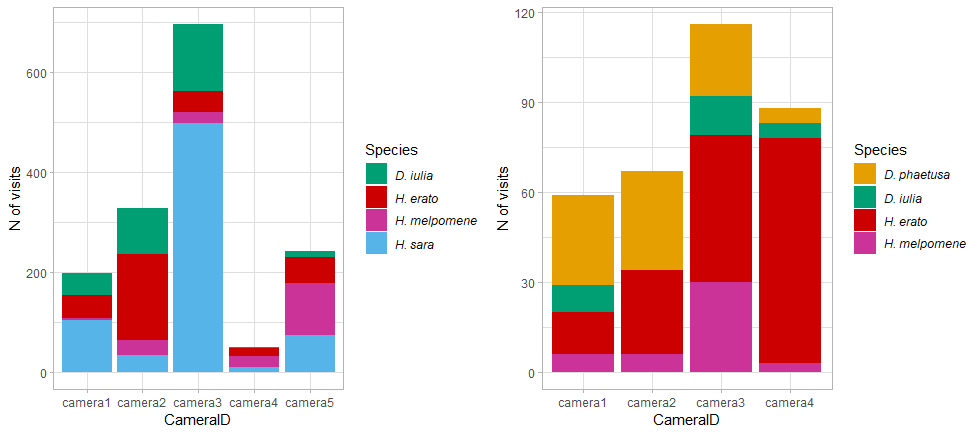


**Supplementary Figure 2.** Total number of visits in each flower cluster during the first (left) and second (right) trial.

**Supplementary Table 1.** Number of recordings in each camera for each individual during trial 1 and 2.

|  |  |  |  |  |  |  |  |  |
| --- | --- | --- | --- | --- | --- | --- | --- | --- |
| Trial | Species | Ind_ID | Sex | camera1 | camera2 | camera3 | camera4 | camera5 |
| 1 | sara | 1 | M | 0 | 0 | 59 | 0 | 2 |
| 1 | erato | 2 | M | 1 | 2 | 3 | 0 | 8 |
| 1 | erato | 3 | M | 8 | 1 | 1 | 1 | 10 |
| 1 | erato | 4 | M | 0 | 1 | 0 | 0 | 5 |
| 1 | sara | 5 | F | 25 | 2 | 0 | 0 | 0 |
| 1 | dryas | 6 | M | 7 | 4 | 7 | 0 | 7 |
| 1 | sara | 7 | M | 53 | 0 | 2 | 0 | 0 |
| 1 | erato | 8 | M | 1 | 7 | 0 | 1 | 2 |
| 1 | dryas | 9 | M | 1 | 0 | 27 | 0 | 0 |
| 1 | sara | 10 | M | 0 | 0 | 137 | 0 | 0 |
| 1 | erato | 12 | F | 21 | 71 | 2 | 3 | 1 |
| 1 | sara | 14 | F | 0 | 0 | 0 | 0 | 2 |
| 1 | sara | 18 | F | 0 | 6 | 63 | 5 | 0 |
| 1 | sara | 19 | M | 0 | 2 | 45 | 0 | 1 |
| 1 | erato | 20 | M | 0 | 0 | 0 | 1 | 1 |
| 1 | sara | 21 | M | 0 | 0 | 11 | 0 | 49 |
| 1 | sara | 23 | M | 0 | 17 | 117 | 4 | 1 |
| 1 | melpomene | 24 | M | 2 | 4 | 2 | 5 | 20 |
| 1 | melpomene | 25 | F | 0 | 14 | 2 | 0 | 4 |
| 1 | melpomene | 26 | M | 0 | 1 | 0 | 0 | 44 |
| 1 | melpomene | 27 | F | 0 | 3 | 1 | 0 | 0 |
| 1 | dryas | 29 | F | 0 | 0 | 1 | 0 | 0 |
| 1 | erato | 30 | F | 7 | 45 | 1 | 4 | 3 |
| 1 | erato | 31 | M | 2 | 22 | 19 | 0 | 0 |
| 1 | sara | 32 | M | 7 | 2 | 0 | 0 | 19 |
| 1 | dryas | 33 | M | 1 | 6 | 17 | 0 | 0 |
| 1 | erato | 34 | F | 6 | 4 | 1 | 0 | 0 |
| 1 | erato | 36 | F | 0 | 2 | 4 | 7 | 12 |
| 1 | erato | 37 | M | 0 | 15 | 1 | 0 | 5 |
| 1 | dryas | 38 | M | 0 | 0 | 0 | 0 | 1 |
| 1 | dryas | 40 | M | 30 | 74 | 38 | 0 | 2 |
| 1 | dryas | 41 | M | 0 | 1 | 0 | 1 | 1 |
| 1 | melpomene | 42 | M | 0 | 1 | 4 | 1 | 33 |
| 1 | melpomene | 43 | F | 0 | 0 | 0 | 0 | 1 |
| 1 | dryas | 44 | M | 1 | 0 | 37 | 0 | 0 |
| 1 | sara | 45 | M | 0 | 1 | 63 | 0 | 0 |
| 1 | erato | 47 | F | 0 | 0 | 0 | 0 | 2 |
| 1 | dryas | 49 | M | 4 | 1 | 0 | 0 | 1 |
| 1 | erato | 50 | M | 1 | 0 | 0 | 0 | 0 |
| 1 | melpomene | 52 | M | 0 | 0 | 10 | 8 | 0 |
| 1 | melpomene | 54 | M | 0 | 1 | 0 | 5 | 0 |
| 1 | erato | 55 | F | 0 | 2 | 0 | 0 | 0 |
| 1 | melpomene | 56 | M | 0 | 7 | 1 | 2 | 1 |
| 1 | melpomene | 57 | M | 0 | 0 | 1 | 0 | 0 |
| 1 | melpomene | 58 | F | 0 | 0 | 0 | 1 | 0 |
| 1 | melpomene | 59 | F | 1 | 0 | 0 | 0 | 0 |
| 1 | sara | 62 | F | 19 | 3 | 0 | 0 | 0 |
| 1 | dryas | 63 | M | 0 | 4 | 1 | 0 | 0 |
| 1 | erato | 64 | M | 0 | 0 | 11 | 0 | 0 |
| 1 | dryas | 65 | M | 0 | 2 | 5 | 0 | 1 |
| 1 | erato | 66 | F | 0 | 0 | 0 | 0 | 3 |
| 2 | dryadula | dd1 | M | 0 | 15 | 11 | 0 | - |
| 2 | dryadula | dd10 | M | 10 | 0 | 0 | 0 | - |
| 2 | dryadula | dd3 | M | 6 | 6 | 7 | 0 | - |
| 2 | dryadula | dd4 | M | 14 | 7 | 4 | 4 | - |
| 2 | dryadula | dd5 | M | 0 | 5 | 2 | 0 | - |
| 2 | dryadula | dd6 | F | 0 | 0 | 0 | 1 | - |
| 2 | dryas | d1 | F | 3 | 0 | 0 | 0 | - |
| 2 | dryas | d2 | M | 4 | 0 | 6 | 2 | - |
| 2 | dryas | d3 | M | 2 | 0 | 3 | 1 | - |
| 2 | dryas | d4 | M | 0 | 0 | 3 | 2 | - |
| 2 | dryas | d8 | M | 0 | 0 | 1 | 0 | - |
| 2 | erato | 1e | F | 0 | 0 | 4 | 18 | - |
| 2 | erato | 1ee | F | 0 | 3 | 1 | 0 | - |
| 2 | erato | 2e | F | 0 | 0 | 7 | 11 | - |
| 2 | erato | 2ee | M | 0 | 1 | 0 | 0 | - |
| 2 | erato | 3e | M | 0 | 0 | 0 | 1 | - |
| 2 | erato | 3ee | F | 1 | 4 | 9 | 20 | - |
| 2 | erato | 4ee | F | 4 | 0 | 2 | 0 | - |
| 2 | erato | 5e | F | 0 | 0 | 9 | 0 | - |
| 2 | erato | 5ee | F | 1 | 0 | 1 | 1 | - |
| 2 | erato | 6ee | F | 3 | 15 | 0 | 12 | - |
| 2 | erato | 7ee | M | 1 | 0 | 11 | 6 | - |
| 2 | erato | 8ee | F | 2 | 1 | 4 | 5 | - |
| 2 | erato | e11 | F | 0 | 2 | 1 | 0 | - |
| 2 | erato | e7 | M | 0 | 2 | 0 | 0 | - |
| 2 | erato | e9 | F | 2 | 0 | 0 | 1 | - |
| 2 | melpomene | m11 | M | 0 | 0 | 1 | 2 | - |
| 2 | melpomene | m12 | F | 0 | 0 | 4 | 0 | - |
| 2 | melpomene | m13 | F | 0 | 0 | 6 | 0 | - |
| 2 | melpomene | m2 | F | 0 | 5 | 3 | 0 | - |
| 2 | melpomene | m3 | F | 0 | 0 | 1 | 0 | - |
| 2 | melpomene | m4 | M | 3 | 1 | 2 | 0 | - |
| 2 | melpomene | m7 | F | 0 | 0 | 10 | 0 | - |
| 2 | melpomene | m8 | F | 3 | 0 | 0 | 0 | - |
| 2 | melpomene | m9 | F | 0 | 0 | 3 | 1 | - |
|  |  |  |  |  |  |  |  |  |

**Supplemental Table 2.** Qui-square results for each individual during trial 1 and 2.

| Trial | Species | Ind_ID | X^2^ | df | *p*_value |
| --- | --- | --- | --- | --- | --- |
| 1 | sara | 1 | 224.6 | 4 | 2.2E-16 |
| 1 | erato | 2 | 13.85 | 4 | 0.007765 |
| 1 | erato | 3 | 18.76 | 4 | 0.000875 |
| 1 | erato | 4 | 15.66 | 4 | 0.003501 |
| 1 | sara | 5 | 89.48 | 4 | 2.2E-16 |
| 1 | dryas | 6 | 7.6 | 4 | 0.1074 |
| 1 | sara | 7 | 200.73 | 4 | 2.2E-16 |
| 1 | erato | 8 | 14 | 4 | 0.007295 |
| 1 | dryas | 9 | 102.36 | 4 | 2.2E-16 |
| 1 | sara | 10 | 548 | 4 | 2.2E-16 |
| 1 | erato | 12 | 182.41 | 4 | 2.2E-16 |
| 1 | sara | 14 | 8 | 4 | 0.09158 |
| 1 | sara | 18 | 198.3 | 4 | 2.2E-16 |
| 1 | sara | 19 | 163.46 | 4 | 2.2E-16 |
| 1 | erato | 20 | 3 | 4 | 0.5578 |
| 1 | sara | 21 | 150.17 | 4 | 2.2E-16 |
| 1 | sara | 23 | 364.42 | 4 | 2.2E-16 |
| 1 | melpomene | 24 | 35.03 | 4 | 4.58E-07 |
| 1 | melpomene | 25 | 34 | 4 | 7.45E-07 |
| 1 | melpomene | 26 | 170.22 | 4 | 2.2E-16 |
| 1 | melpomene | 27 | 8.5 | 4 | 0.07489 |
| 1 | dryas | 29 | 4 | 4 | 0.406 |
| 1 | erato | 30 | 115 | 4 | 2.2E-16 |
| 1 | erato | 31 | 55.72 | 4 | 2.29E-11 |
| 1 | sara | 32 | 45.92 | 4 | 2.55E-09 |
| 1 | dryas | 33 | 43.91 | 4 | 6.68E-09 |
| 1 | erato | 34 | 13.09 | 4 | 0.01084 |
| 1 | erato | 36 | 17.6 | 4 | 0.001477 |
| 1 | erato | 37 | 38.76 | 4 | 7.8E-08 |
| 1 | dryas | 38 | 4 | 4 | 0.406 |
| 1 | dryas | 40 | 127.67 | 4 | 2.2E-16 |
| 1 | dryas | 41 | 2 | 4 | 0.7358 |
| 1 | melpomene | 42 | 102.92 | 4 | 2.2E-16 |
| 1 | melpomene | 43 | 4 | 4 | 0.406 |
| 1 | dryas | 44 | 142.26 | 4 | 2.2E-16 |
| 1 | sara | 45 | 246.16 | 4 | 2.2E-16 |
| 1 | erato | 47 | 8 | 4 | 0.09158 |
| 1 | dryas | 49 | 9 | 4 | 0.0611 |
| 1 | erato | 50 | 4 | 4 | 0.406 |
| 1 | melpomene | 52 | 27.55 | 4 | 1.54E-05 |
| 1 | melpomene | 54 | 15.66 | 4 | 0.003501 |
| 1 | erato | 55 | 8 | 4 | 0.09158 |
| 1 | melpomene | 56 | 14 | 4 | 0.007295 |
| 1 | melpomene | 57 | 4 | 4 | 0.406 |
| 1 | melpomene | 58 | 4 | 4 | 0.406 |
| 1 | melpomene | 59 | 4 | 4 | 0.406 |
| 1 | sara | 62 | 62.09 | 4 | 1.05E-12 |
| 1 | dryas | 63 | 12 | 4 | 0.01735 |
| 1 | erato | 64 | 44 | 4 | 6.42E-09 |
| 1 | dryas | 65 | 10.75 | 4 | 0.02952 |
| 1 | erato | 66 | 12 | 4 | 0.01735 |
| 2 | dryadula | dd1 | 27.23 | 3 | 5.27E-06 |
| 2 | dryadula | dd10 | 30 | 3 | 1.38E-06 |
| 2 | dryadula | dd3 | 6.47 | 3 | 0.09071 |
| 2 | dryadula | dd4 | 9.2 | 3 | 0.02666 |
| 2 | dryadula | dd5 | 9.5 | 3 | 0.02258 |
| 2 | dryadula | dd6 | 3 | 3 | 0.3916 |
| 2 | dryas | d1 | 9 | 3 | 0.02929 |
| 2 | dryas | d2 | 6.66 | 3 | 0.08332 |
| 2 | dryas | d3 | 3.33 | 3 | 0.343 |
| 2 | dryas | d4 | 5.4 | 3 | 0.1447 |
| 2 | dryas | d8 | 3 | 3 | 0.3916 |
| 2 | erato | 1e | 39.8 | 3 | 1.16E-08 |
| 2 | erato | 1ee | 6 | 3 | 0.1116 |
| 2 | erato | 2e | 19.77 | 3 | 0.000189 |
| 2 | erato | 2ee | 3 | 3 | 0.3916 |
| 2 | erato | 3e | 3 | 3 | 0.3916 |
| 2 | erato | 3ee | 24.58 | 3 | 1.88E-05 |
| 2 | erato | 4ee | 7.3 | 3 | 0.062 |
| 2 | erato | 5e | 27 | 3 | 5.89E-06 |
| 2 | erato | 5ee | 1 | 3 | 0.8013 |
| 2 | erato | 6ee | 20.4 | 3 | 0.00014 |
| 2 | erato | 7ee | 17.11 | 3 | 0.000671 |
| 2 | erato | 8ee | 3.33 | 3 | 0.343 |
| 2 | erato | e11 | 3.66 | 3 | 0.299 |
| 2 | erato | e7 | 6 | 3 | 0.111 |
| 2 | erato | e9 | 3.66 | 3 | 0.299 |
| 2 | melpomene | m11 | 3.66 | 3 | 0.299 |
| 2 | melpomene | m12 | 12 | 3 | 0.007383 |
| 2 | melpomene | m13 | 18 | 3 | 0.00044 |
| 2 | melpomene | m2 | 9 | 3 | 0.02929 |
| 2 | melpomene | m3 | 3 | 3 | 0.3916 |
| 2 | melpomene | m4 | 3.33 | 3 | 0.343 |
| 2 | melpomene | m7 | 30 | 3 | 1.38E-06 |
| 2 | melpomene | m8 | 9 | 3 | 0.02929 |
| 2 | melpomene | m9 | 6 | 3 | 0.1116 |
